# Massive Open Online Courses on Health and Medicine: Review

**DOI:** 10.2196/jmir.3439

**Published:** 2014-08-14

**Authors:** Tharindu Rekha Liyanagunawardena, Shirley Ann Williams

**Affiliations:** ^1^School of Systems EngineeringUniversity of ReadingReadingUnited Kingdom

**Keywords:** eLearning, education, health education, continuing education, computer-assisted instruction

## Abstract

**Background:**

Massive open online courses (MOOCs) have become immensely popular in a short span of time. However, there is very little research exploring MOOCs in the discipline of health and medicine.

**Objective:**

We aim to provide a review of MOOCs related to health and medicine offered by various MOOC platforms in 2013, by analyzing and comparing the various offerings, their target audience, typical length of course, and credentials offered. We also discuss opportunities and challenges presented by MOOCs in health and medicine.

**Methods:**

Health and medicine–related MOOCs were gathered using several methods to ensure the richness and completeness of data. Identified MOOC platform websites were used to gather the lists of offerings. In parallel, these MOOC platforms were contacted to access official data on their offerings. Two MOOC aggregator sites (Class Central and MOOC List) were also consulted to gather data on MOOC offerings. Eligibility criteria were defined to concentrate on the courses that were offered in 2013 and primarily on the subject of health and medicine. All language translations in this paper were done using Google Translate.

**Results:**

The search identified 225 courses, of which 98 were eligible for the review. Over half (58%, 57/98) of the MOOCs considered were offered on the Coursera platform, and 94% (92/98) of all the MOOCs were offered in English. Universities offered 90 MOOCs, and the John Hopkins University offered the largest number of MOOCs (12/90). Only three MOOCs were offered by developing countries (China, West Indies, and Saudi Arabia). The duration of MOOCs varied from 3-20 weeks with an average length of 6.7 weeks. On average, MOOCs expected a participant to work on the material for 4.2 hours a week. Verified certificates were offered by 14 MOOCs, while three others offered other professional recognition.

**Conclusions:**

The review presents evidence to suggest that MOOCs can be used as a way to provide continuous medical education. It also shows the potential of MOOCs as a means of increasing health literacy among the public.

##  Introduction

### Background

Massive open online courses (MOOCs) are a recent innovative addition to the online learning landscape. They are online courses that are accessible through the Web and open to registration generally without limits on numbers or prerequisites. The course registration and course materials are free of charge, although in some courses one can pay to obtain a certificate of participation or verified certificate (for credit). These courses have start and end dates, but even after the start date, registration is often kept open unlike traditional online courses that close registration at the start of the course. MOOCs carry great potential to reach large numbers of learners from across the world as they can be accessed by anyone anywhere in the world as long as they have Internet access, computer literacy, and language proficiency.

### Brief History

“Connectivism and Connective Knowledge” (CCK08), an online course facilitated by George Siemens and Stephen Downes, in 2008, offered through the Learning Technologies Centre and Extended Education at the University of Manitoba [1], is considered the first MOOC [2]. This online course had around 2200 non-credit, non-fee paying students along with 25 paid enrollments (for credit). Unlike traditional online courses that rely primarily on resources posted by the facilitators through a learning management system, this course was conducted according to the principles of connectivism [3], encouraging learning through a network (peer learning) across multiple learning spaces.

Within a short time, MOOCs have attracted wide interest from educators, learners, businesses, media, and the general public. Many prominent universities are now offering their courses as MOOCs. For example, Massachusetts Institute of Technology, Harvard University, Berkeley University of California, and the University of Texas offer MOOCs through the MOOC platform edX. There have also been for-profit ventures such as the Coursera MOOC platform, which partners with over 100 institutions (108 as of March 19, 2014) from around the world.

In some MOOCs, there are hundreds of thousands of enrollments. However, not all students enrolled return for the course and only a small number of them finish all parts of the course [2]. Given the nature of the courses, where participation is voluntary and no financial commitments are made up front, is the number of students who complete the course a concern? If the aim of a MOOC is to provide the opportunity or access to learn from high-quality courses (taught by the experts in the field from world class universities), then the numbers completing the course should not be of prime concern [4]. On the other hand, if the aim were to get everyone registered through to the end, similar to a traditional higher educational institution where a student failing to complete within a given timeframe could adversely affect the university’s profile, family, student, and lecturers [5], these completion rates would be a disaster. The problem here could be the use of traditional metrics in this non-traditional or disruptive form of educational provision. However, more evidence-based research may reveal the true nature of MOOCs and possibly better ways of understanding and evaluating them.

Although the MOOC revolution began in North America, it has now spread to universities and institutions in many parts of the developed world. For example, in 2013, the UK MOOC platform, FutureLearn, started offering courses. Initially MOOCs were offered in English, but today there are many MOOCs offered in various languages including Chinese, Arabic, Spanish, and French. For example, in 2012, a Spanish MOOC platform Miriada X was founded, and in 2013, the platform Rawq started offering courses in Arabic. Similarly, XuetangX was created to offer courses in Chinese. However, English remains the dominant language in MOOC provision.

### Pedagogy

MOOCs, like other online courses, use a variety of learning materials including videos, documents, and quizzes. At present, MOOCs are mainly classified according to their pedagogical position: connectivist MOOCs (cMOOCs) and “MOOC as eXtension of something else” (xMOOCs) [6]. cMOOCs harness the strength of networks and peer learning generally using multiple learning spaces. Participants in cMOOCs are likely to find a lot of emphasis on participants’ stories and learning from them (eg, Rhizomatic Learning: The community is the curriculum on P2PUniversity) than on the learning materials provided by the instructor or course designer. On the other hand, xMOOCs seems to have a more individualist learning approach [7] surrounding the course on a given MOOC platform. In xMOOCs, learning and understanding the content provided in the course is given priority. Original cMOOCs were based on open education practices making their content available using open licensing [8]. However, many xMOOCs offered in platforms such as Coursera use copyrighted materials. However, it is worthwhile noting that there is a continuum of possibilities between these two distinct pedagogical positions.

MOOCs are offered in a wide range of subjects varying from cell biology to astronomy. In this paper, we explore the courses offered by major MOOC platforms on topics related to health and medicine. Several methods were used to collect relevant courses for the review: directly making contact with MOOC platforms to get course data, accessing publicly available information on MOOC platform websites, and using MOOC aggregator sites. Data related to courses offered in 2013 that were collected as earlier offerings lacked relevant details. The paper provides a comprehensive review of MOOCs offered in 2013 in “Health and Medicine” or a related category.

##  Methods

### Data Collection

#### Overview

In general, researchers use different methods to identify data to be included in a review. For example, to collect papers (data) for a systematic review of literature, researchers would search in databases and/or search engines and chaining from known sources [2]. Similarly, in collecting details of MOOCs offered in topics related to health and medicine for this review, it was important to collect as complete a set of data as possible. A list of MOOCs offered by various providers was not readily available for analysis. Thus, in identifying relevant MOOCs, a range of methods were used to obtain related information that would form a more complete dataset for the analysis.

#### Platforms

With the growing popularity of MOOCs, there have been various commercial and non-commercial organizations providing platforms where MOOCs can be offered. Identification of such MOOC platforms was carried out using the literature, news items, and Web resources. LISTedTECH (a database of educational companies, educational products, and educational institutions that anyone can edit) lists 19 systems as MOOC platforms as of December 19, 2013 [9]. Using news articles, blogs, and other literature, nine additional MOOC platforms that are in operation were identified. The total of 28 identified MOOC platforms (see Multimedia Appendix 1) and their offerings were considered in this review.

From December 17-21, 2013, each of these MOOC platforms’ websites were accessed to find the list of MOOCs offered by each of them on topics relating to health and medicine. In instances where the websites were in languages other than English, Google Translate was used.

#### Official Records

In parallel, MOOC platform providers were contacted via email to obtain official records when their websites did not have the necessary information. Only five MOOC platform providers (Canvas, iversity, Openlearning, Miriada X, and Crypt4you) responded to this request with information while another MOOC provider (Coursera) responded without the information.

#### Aggregators

The two MOOC aggregator sites Class Central [10] and MOOC List [11] were also consulted to collect a list of MOOCs.

### Eligibility

#### Free Courses

When platforms provided both paid-for and free courses (such as Udemy), only free courses were considered. Courses offered by University of Miami Global Academy required a US$90 non-refundable one-time registration fee upfront and a tuition fee depending on the number of credits taken. Thus, courses offered by this platform were not included in this review.

#### Subject

MOOCs listed under “Health and Medicine” or a related category (such as Health Sciences on Miriada X, Health Science on CourseSites, Health and Society on Coursera) were considered. When MOOCs were not categorized (such as OpenupEd and FutureLearn), the course title and where available the course description were used to determine if it was related to health and medicine (eg, “Improving your image: Dental Photography in Practice” on FutureLearn).

MOOCs categorized under Psychology or Biology and Life Sciences (or were predominantly on them) were not considered in this analysis. MOOCs on veterinary sciences but categorized under Health and Medicine (eg, “Canine Theriogenology for Dog Enthusiasts” on Coursera) were also excluded. But when the courses discussed animal health or diseases and their impact/influence on human health, such as “Enfermedades transfronterizas de los animals” (Animal transboundary diseases) on Miriada X, they were included.

#### Start Date

The time period for the review was defined as January 1 to December 31, 2013 (inclusive). MOOCs having a start date within this period were considered for the review. Self-paced MOOCs (that do not have a specified start date) were omitted. This included 39 courses listed under Health and Medicine in the Veduca platform, 10 courses listed under Health and Fitness in the Udemy platform, and 44 courses listed under Health Literacy on the ALISON platform, and four OpenupEd courses (“Stress post-traumatic disorder: difficulties and debate in making a diagnosis”, “Valutazione clinica e strumenti di indagine nell'area traumatica”[Clinical assessment and survey instruments in traumatic area], “Programmi e modelli di intervento nelle situazioni traumatiche” [Programs and intervention models in traumatic situations], and “Anatomo-physiological bases of mental activity”). On the Saylor platform, all courses are self-paced (the titles that seemed relevant were categorized under Biology). The course “La Seguridad del Paciente” (Patient Safety) on Miriada X had to be discounted because the start date for the course could not be established.

#### Class Central

We found 113 MOOCs related to health and medicine listed in the MOOC aggregator site Class Central [10]. Under the “Finished Courses” section, exactly 100 courses were listed, while 13 were listed in the “Courses in Progress” section (December 24, 2013). A number of courses were excluded for a variety of reasons.

Five courses were excluded from “Courses in Progress”:

- “Exploring anatomy: the human abdomen” offered by the University of Leeds on the FutureLearn platform had an incorrect start date in 2013 instead of the correct start date in 2014
- Three self-paced MOOCs (“The Basics of Exercise Programs for Older Adults” on CourseSites, two Stanford University offerings “Practical tips to improve Asian American participation in cancer clinical trials”, and “Antimicrobial Stewardship: Optimization of Antibiotic Practices”, each 104 weeks long)
- “DEV: Water, Civilization, and Nature: Addressing 21st Century Water Challenges” on CourseSites, which was a self-paced course lacking relevance

We excluded 19 courses from “Finished Courses”:

- Nine courses offered in 2012
- Two courses without start dates (“Cardiac Arrest, Hypothermia, and Resuscitation Science” and “Basic Behavioral Neurology” offered by University of Pennsylvania on Coursera).
- Eight courses lacking relevance: “Marathon Training” and “Safety Function & Action: Strategies for Disaster Responders” on Canvas.net, “Critical Thinking in Global Changes” offered by the University of Edinburgh, “Canine Theriogenology for Dog Enthusiasts” offered by University of Minnesota (2 instances), “Equine Nutrition” offered by University of Edinburgh, “Growing Old Around the Globe” offered by the University of Pennsylvania, and “Disaster Preparedness” offered by the University of Pittsburgh on Coursera

The “Understanding Dementia” MOOC was offered by University of Tasmania on Desire2Learn. Desire2Learn was not listed as a MOOC platform as it offered only proprietary software for institutions. But the MOOC was included in the review. Thus a list of 89 relevant MOOCs (out of 113 identified) was obtained from the Class Central aggregator site.

#### MOOC List

Another MOOC aggregator site, MOOC List [11], listed details of 54 MOOCs in 2013 under “Health and Society” and 45 under “Medicine and Pharmacology” (January 3, 2014). Due to 19 overlapping courses in the two categories, the distinct course count was 80. Out of these, 53 courses overlapped with the list obtained through Class Central, which left a list of 27 new courses. We further disregarded some courses:

- Four self-paced courses: “Bioethics” and “Make the Strategic Case for Disability in the Workplace” on Canvas; “Clinical Psychology” on Saylor and “Enseñanza en consulta y medio hospitalario” (Education in consultation and hospital environment) on CourseSites
- “Introduction to Pharmaceutical Manufacturing” offered by Dublin Institute of Technology on CourseSites with a November 25 start date could not be validated against the MOOC list available from the official website (there was a MOOC “So you want to work in the pharmaceutical industry?... Next Steps” offered by Dublin Institute of Technology on CourseSites and authors believe this entry was thus erroneous)
- 13 courses lacking relevance

**Figure 1:**
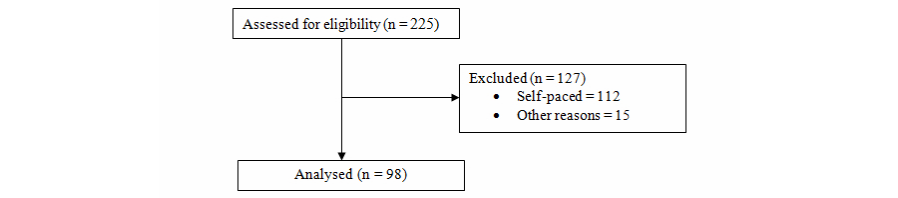
Flow diagram.

This led to nine entries (seven Coursera courses, a P2P University course, and a course offered by Stanford University VentureLab) from MOOC List being added to the Class Central list (of 89 entries). Therefore, the total number of MOOCs considered for this review is 98 (see Figure 1). The collection of MOOCs included in the review is given in Multimedia Appendix 2. The number of MOOCs from each platform considered in this review is given in Table 1.

**Table 1:** Number of MOOCs included in the review per platform.

Platform		Total found (N=225)	Self-paced (n=112)	Excluded other reasons (n=15)	Considered for review (n=98)	Not considered for review (n=127)
1	ALISON	44	44		0	44
2	Canvas.net	9	2	2	5	4
3	Coursera	67	2	8	57	10
4	CourseSites	11	3	1	7	4
5	Coursolve	0			0	
6	Crypt4you	0			0	
7	edX	7		2	5^a^	2
8	France Universite Numerique	0			0	
9	FutureLearn	2		1	1	1
10	Galileo Education Systems	0			0	
11	Rwaq	1			1	
12	Iversity	0			0	
13	Miriada X	5		1	4	1
14	NovoEd	1			1	
15	OpenLearning	1	1		0	1
16	Open2Study	14			14	
17	OpenHPI	0			0	
18	OpenupEd	4	4		0	4
19	P2PUniversity	1			1	
20	Saylor	7	7		0	7
21	Skynet	0			0	
22	Udacity	0			0	
23	Udemy	10	10		0	10
24	uneopen.com	0			0	
25	UKeU (not in operation)	0			0	
26	University of Miami Global	0			0	
27	Veduca	39	39		0	39
28	XuetangX	0			0	
29	Stanford University VentureLab	1			1	
30	University of Tasmania on Desire2Learn	1			1	

^a^A course offered by Stanford University in OpenEdX was also considered as edX.

## Results

### Massive Open Online Course Platforms

**Figure 2:**
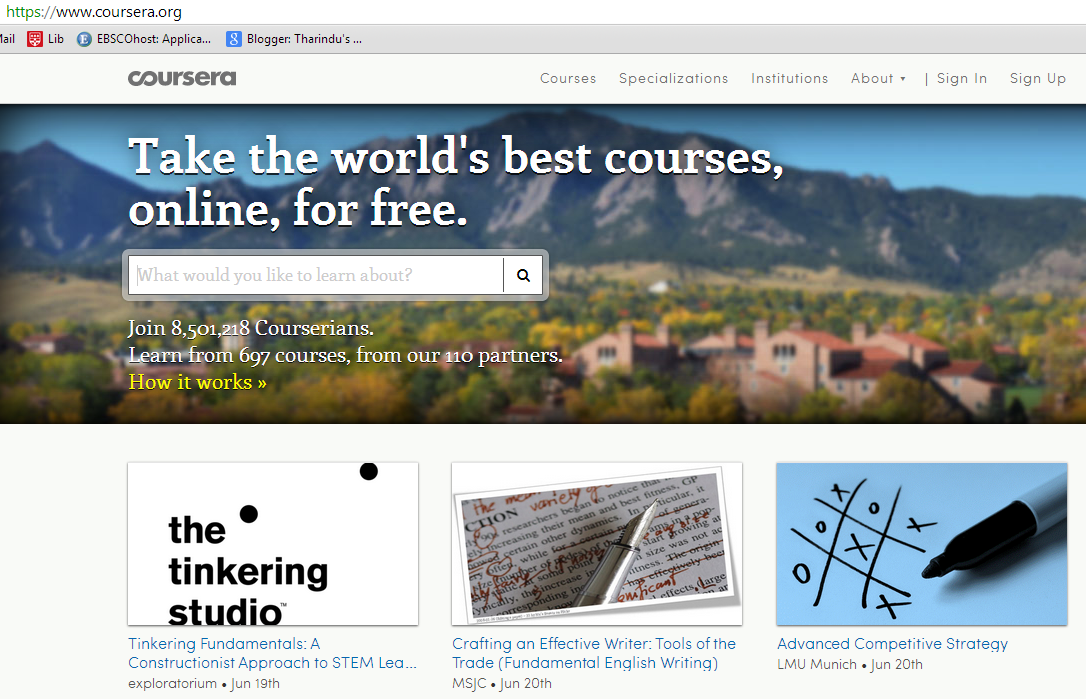
Coursera platform.

**Figure 3:**
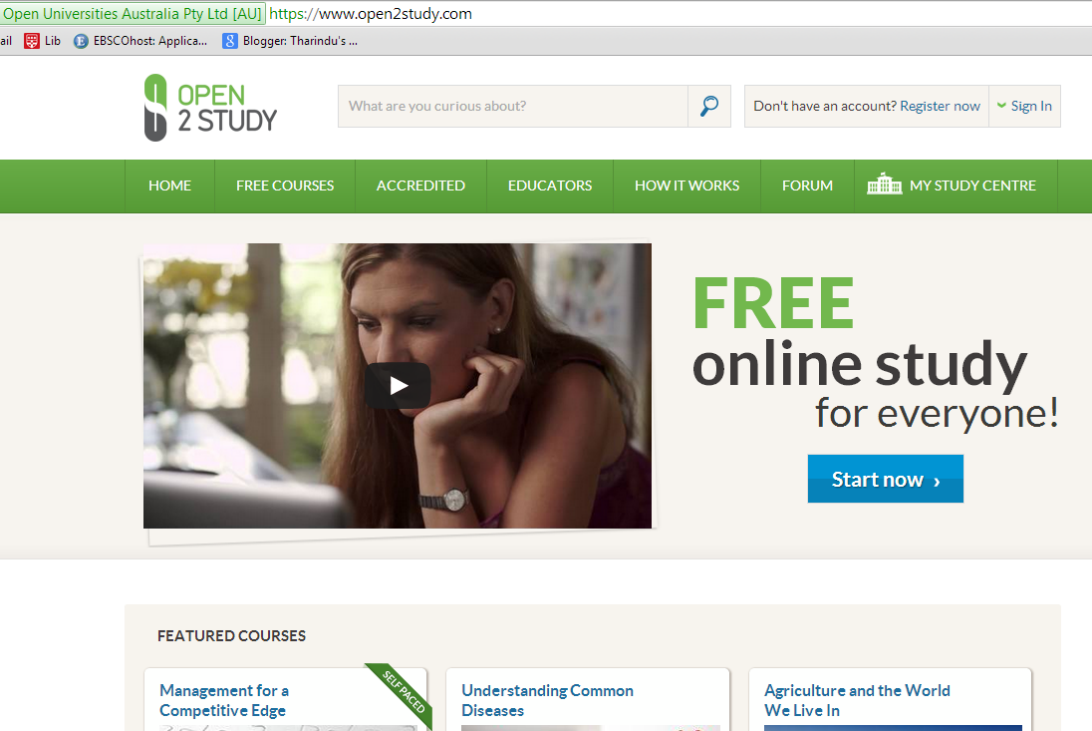
Open2Study platform.

Quantitative and qualitative analyses of the data were performed using Microsoft Excel and NVivo software. The majority (58%, 57/98) of MOOCs related to health and medicine was offered by Coursera (Figure 2) followed by Open2Study (Figure 3). Full analysis of course offerings by platform/provider is shown in Table 2.

**Table 2:** MOOCs by platform/provider (n=98).

Platform/provider	n	%
Coursera	57	58
Open2Study	14	14
CourseSites	7	7
Canvas	5	5
edX	5	5
Miriada X	4	4
FutureLearn	1	1
NovoEd	1	1
P2PUniversity	1	1
Rwaq	1	1
University of Tasmania	1	1
VentureLab	1	1

### Language

The language breakdown of MOOCs related to health and medicine shows that the vast majority of MOOCs, 94% (92/98), were offered in English (Table 3). Four MOOCs were offered in Spanish (Castilian) on Miriada X, while one MOOC each was offered in Arabic on Rwaq and in Chinese on Coursera.

**Table 3:** MOOCs by language (n=98).

Language	n	%
English	92	94
Spanish	4	4
Arabic	1	1
Chinese	1	1

### Offering Institution

The highest number of MOOCs in this review were offered by John Hopkins University (12) followed by University of California (nine), University of Pennsylvania (seven), and Open Universities Australia (six). Harvard University and the University of Sheffield offered three MOOCs each.

Most of the MOOCs (90/98) in the review were offered by universities. The large majority of these MOOCs, 70% (63/90) were offered by North American universities. Out of these, only two MOOCs were offered by Canadian universities (University of Toronto). Universities in the other parts of the world offered only a small number of MOOCs in health and medicine (Table 4). These MOOCs were offered by 14 universities (Table 5): five in Australia, four in Spain, two in the United Kingdom, one in each of the Republic of Ireland, Denmark, Switzerland, the West Indies, and China. Also considering the “Introduction to Psychiatry” MOOC offered on Rwaq, very few MOOCs (3/98, 3%) were from developing countries.

**Table 4:** MOOCs by North American universities versus worldwide (n=90).

	n	%
North America	63	70
Other	27	30

**Table 5:** MOOCs offered by universities outside North America (n=27).

University	MOOCs, n
Open Universities Australia	6
Dublin Institute of Technology	3
University of Sheffield	3
University of Copenhagen	2
Flinders University	2
The University of Melbourne	1
University of Geneva	1
Shanghai Jiao Tong University	1
University of Birmingham	1
Universidad De Murcia	1
Universidad De Cantabria	1
Universitat Plitecnica De Valencia	1
CEU Universidad San Pablo	1
University of Wollongong	1
University of Tasmania	1
St. George’s University, Grenada	1

### Number of Instances

Many MOOCs have run only one instance within the considered period. However, the MOOCs “Food, Nutrition and Your Health” and “Introduction to Nursing in Healthcare” both offered by Open2Study have both run six times. The MOOCs that were offered more than once are listed in Table 6.

**Table 6:** MOOCs offered multiple times.

MOOC	Platform	Instances, n
Food, Nutrition and Your Health	Open2Study	6
Introduction to Nursing in Healthcare	Open2Study	6
Health for All through Primary Care	Coursera	3
Contraception: Choices, Culture and Consequences	Coursera	2
The Social Context of Mental Health and Illness	Coursera	2
Genes and the Human Condition (From Behavior to Biotechnology)	Coursera	2
Nutrition for Health Promotion and Disease Prevention	Coursera	2
Health Informatics in the Cloud	Coursera	2
So you want to work in the Pharmaceutical Industry	CourseSites	2

### Duration

The length of the MOOCs considered for the review varied from 3 weeks (“Introduction to Pharmaceutical Manufacturing Technologies” and “So you want to work in the Pharmaceutical Industry”, two instances) to 20 weeks (“International Health Systems”) with a mode of 6 weeks (21 MOOCs) and average length of 6.7 weeks. In calculating the duration of MOOCs, only 96 MOOCs were considered as the duration of two MOOCs could not be verified. Many MOOCs (75) were 8 weeks or less in duration.

### Time Commitment

Most MOOC descriptions (76) contained information on the average time a participant was expected to work on the materials. On average, the MOOCs expected a participant to work on the material for 4.2 hours a week. The Stanford University offering “HRP258: Statistics in Medicine” expected the highest commitment of 8-12 hours per week. Most courses (mode) expected 2-4 hours per week on the course.

### Recognition

Some of the MOOCs considered in the review provided certificates for successful participants. The terminology used in different platforms to refer to certificates varied. For example, in Coursera, a “statement of accomplishment” referred to the free certificate signed by the instructor or educator (professor), while in edX a similar credential was referred to as an “honor code certificate”. On Mirianda X, the free certificate was referred to as “certificados de participación” (certificate of participation) and the paid-for certificate was referred to as “certificado de superación” (certificate of overcoming).

According to course descriptions, the Stanford University course “HRP258: Statistics in Medicine” offered a certificate of participation to students who obtained 60% or higher and a certificate with distinction for participants obtaining 90% or higher. Some MOOC descriptions specifically mentioned that the awarded certificate does not carry credits. These were offered by Emory University, the University of Melbourne, University of Geneva, the College of St. Scholastica, and the University of Pennsylvania.

Verified certificates were offered for some courses while some others were eligible for continuous professional development credit. John Hopkins University offered four courses with verified certificates, while the University of Maryland and the Georgia Institute of Technology each offered two courses with verified certification. Other universities that offered verified certification were the College of St. Scholastica, Duke University, Vanderbilt University, HarvardX, CEU Universidad San Pablo, and Universitat Politecnica de Valencia. Only three courses offered other professional qualifications. Two courses (“Care of Elders with Alzheimer’s Disease and other Major Neurocognitive Disorders” and “Global Tuberculosis (TB) Clinical Management and Research”) offered by John Hopkins University were eligible for Continuing Nursing Education (CNE) Credit while “Caries Management by Risk Assessment (CAMBRA)” by University of California offered 12 units of Continuing Dental Education credit for practicing dental professionals and Continuing Medical Education (CME) Credit for practicing physicians. The summary of certification types offered in MOOC descriptions are shown in Table 7.

**Table 7:** Certification types on MOOC descriptions.

Type of certification	n	%^a^
Statement of accomplishment	59	91
Statement of accomplishment mentioning no credit awarded	5	8
Verified certificates	14	22
Other professional recognition	3	5

^a^There were 65 MOOCs that offered at least one type of certification.

### Prerequisites

Out of the considered courses, 59 course descriptions specifically mentioned whether there were prerequisites or not (Table 8). Some courses used “no prerequisites” or “all are welcome” to describe that the course did not have prerequisites, while some others (eg, “Training and Learning Programs for Volunteer Community Health Workers”) mentioned “Some background in community health programs is helpful but not necessary” (these are categorized under “no prerequisite but helpful background”).

**Table 8:** Prerequisites in MOOC descriptions (n=59).

Prerequisites	n	%
No prerequisites	17	29
No prerequisites but helpful background	14	24
With prerequisites	28	47

### Qualitative Analysis

A word frequency analysis (in NVivo) of course titles (titles in other language were translated using Google Translate) showed that the word most frequently used was “Health” with 35 occurrences (Table 9). The next highest used word was “Introduction” with 13 occurrences. Given that 31 MOOCs had no prerequisites to join, this suggests that many courses offered are introductory level.

**Table 9:** Frequently used words in MOOC titles.

Word	Frequency
Health	35
Introduction	13
Nutrition	12
Health care	10
Food	8
Nursing	6
Care	5
Clinical	5
Human	5

### Target Groups

Only 16 courses specified the target audience for the course. A word frequency analysis (in NVivo) of the audience specified showed that the word most frequently used to define target audience was “health” with 10 occurrences followed by the word “professionals” with 8 occurrences.

##  Discussion

### Health Inequality

This review of MOOCs offered in the area of health and medicine during 2013 provides interesting insights, especially the fact that out of the 98 MOOCs only two were offered by universities in developing countries (“One Health One Medicine” by St. George’s University, Grenada, West Indies, and ‘Traditional Chinese Medicine and Chinese Culture” by Shanghai Jiao Tong University, China). This is not unusual, as to date the large majority of MOOCs are offered by institutes in the developed countries. A contributor for this observation specifically in health and medicine-related MOOCs could be the advanced technologies used in prevention, detection, and treatment in the health care systems of the developed countries and their willingness to showcase the success. On the other hand, it can also be an indicator of health inequality between countries. None of the developing countries’ expertise, for example in tropical diseases, is offered as MOOCs. It is noted that open education resources (OERs) on tropical diseases developed by subject experts in Malawi and Ghana from the African Health OER Network [12] are used in the University of Michigan’s medical programs [13]. Similar collaborations with experts from developing countries/universities on MOOCs may create MOOCs that would be of wider interest. The recent edX partnership with Google to jointly develop the edX open source learning platform perhaps will expand the availability of the platform [14] to individuals and institutions.

### Continued Medical Education

Volandes et al [15] argue that online video learning techniques could empower both clinicians and patients. In fact, MOOCs could well be used as a method for Continuing Medical Education (CME). In this review, we found a number of MOOCs that offered verified certification and counted credits toward Continuing Nursing Education, Continuing Dental Education, and CME. Hoy [16] shows that MOOCs can be a convenient and economical method of CME, with the declining industry funding for CME activities.

### Medical Student Education

MOOCs can also provide education to students currently undergoing training to become health professionals. For example, the Coursera course “Clinical Terminology for International and US Students” offered by University of Pittsburgh is aimed at new students in the medical field. Courses such as “Going out on a limb: Anatomy of the upper limb” on Coursera by University of Pennsylvania can supplement traditional medical education or perhaps could even be considered as a “flipped-classroom” [17] experience where the MOOC replaces the lecture and the contact hours with the professor used for a more meaningful discussion.

### Health Literacy

Health literacy is a broad concept with different definitions. Here, we consider it to be “the degree to which people are able to access, understand, appraise and communicate information to engage with the demands of different health contexts in order to promote and maintain good health across the life-course” [18]. Health literacy, similar to literacy is of critical importance for everyday living [19]. It is not just the ability to make sense of health information but is also a strategy for citizenship and empowerment [19,20]. In this information age, eHealth literacy, or “the ability of people to use emerging information and communications technologies to improve or enable health and health care” [21], is becoming even more relevant.

Specialist information on subjects including health and medicine is becoming widely available today. However, information overload and the availability of unreliable information sources on the Internet present a huge challenge for the general public looking for information on a specific medical condition. Availability of MOOCs (especially if the content is open) is likely to help those who are seeking information. As the content is offered by a reputed institution, it becomes easy to identify it as an authentic and credible source.

### Patient Education

MOOCs on health and medicine allow the general public to acquire health education on very specialist topics. One potential area that can be targeted by health and medicine MOOCs is patient education. For example, the MOOC “Care of Elders with Alzheimer’s Disease and other Major Neurocognitive Disorders” provides information to anyone who is interested in knowing about Alzheimer’s disease. This MOOC welcomes patients in early stages of the disease to help them understand the implications of the disease. Participation in these MOOCs is likely to inform patients of their condition and advanced techniques and interventions that are available. It could, to some extent, bridge the language gap (medical terminology) and knowledge gap between patients and doctors. Thus patients would be able to engage in a meaningful discussion with medical professionals on the care they receive and other alternative treatments.

However, in high power distance cultures, this enlightenment of patients may not be well received by health care professionals. In some instances, informed patients or carers may request treatment not yet available in their contexts. On the other hand, informing patients of possible treatments could facilitate medical tourism for those who can afford it.

### Educating the Public

MOOCs can also be used as a tool to educate the public on important health issues. For instance, the Coursera MOOC “Contraception: Choices, Culture and Consequences” offered by University of California educates the public on the importance of reproductive health. Such courses could help people educate themselves without having to talk to a health professional about family planning, which in some cultures is taboo or discouraged by faith. Being able to access trustworthy information through a MOOC could empower people who may otherwise not know the options open to them.

Educating the masses on taboo topics such as “Drug Addiction” could also be achieved with the use of MOOCs. MOOCs generally operate entirely on online spaces; exceptions would be MOOC participants from a locality meeting up for discussions or MOOC participants seeking support from blended provision such as Coursera Learning Hubs [22]. Because one can project a persona in an online space that may differ to who they are in real life, both patients with such conditions and others who seek information can join in, if they wish, without revealing their true identity.

However, suggesting that MOOCs may be a way to educate the public assumes that other conditions for participation in a MOOC (eg, access to technology, skills to use them, and international language competency) are already met. But as Liyanagunawardena, Williams, and Adams [23] show, at present MOOCs may not reach a large proportion of people, especially in developing areas of the world. Current data suggest that a typical course registrant “is a male with a bachelor’s degree who is 26 or older” [24], showing that MOOCs have not yet reached universal accessibility.

### Limitations

This review was conducted by collecting data from various sources. However, as very few MOOC platforms provided official data on their MOOC offerings, only the courses with publicly available course details were used in the review. Collection of data for the review using aggregator sites could have the disadvantage of not including all MOOCs on offer, especially foreign language offerings. By using two aggregator sites and independently collecting data from MOOC platforms, the possibility of this occurring was minimized. In collecting MOOCs for the review, courses categorized under health and medicine or related was considered. However, if a MOOC were wrongly classified, it would not have been captured in the review. In instances where the MOOCs were offered in languages other than English, Google Translate was used to translate the content. Had there been a translation error, it could have affected the data collection process.

### Conclusions

Massive open online courses (MOOCs) have become popular within a short span of time, and there are dozens of providers offering courses in a variety of subjects. Reviewing MOOCs offered on “Health and Medicine” in 2013, we found that 94% of them (92/98) were offered in English and the large majority were offered by North American institutions. Only about 3% of the MOOCs (3/98) were offered by institutions in the developing world. Many courses offered were introductory level. Some courses offered credit toward continuous professional development of medical professionals and verified certificates for a fee, while others offered a statement of participation for successful participants.

There is potential to use MOOCs to educate health care practitioners and students; for example, in continuous professional development of health care professionals. Because they can reach massive numbers across the globe, MOOCs can provide an enormous boost in educating the public on health and medicine, especially on taboo subjects such as acquired immunodeficiency syndrome (AIDS), tuberculosis, and contraception. However, in order to unleash this great potential of MOOCs in educating masses around the globe on health and wellbeing, there are various challenges to overcome (eg, access: language access, physical access to technology, skills access to use technology). Health literacy is a powerful tool that empowers people, and MOOCs could be used to educate the general public to increase their health literacy. The wide variety of MOOCs on various subjects relating to health and medicine offered in 2013 show a glimpse of what is achievable through MOOCs in this discipline.

## Multimedia Appendix 1

MOOC platforms.

## Multimedia Appendix 2

List of MOOCs considered in review.

## Abbreviations

CME: Continued Medical Education
cMOOC: connectivist MOOC
MOOC: massive open online course
OER: Open Educational Resources
xMOOC: MOOC as eXtension of something else
