# Supplementary material for: Massive Open Online Courses on Health and Medicine: Review
Source: J Med Internet Res. 2014 Aug 14;16(8):e191. doi: 10.2196/jmir.3439 (PMC4155756; doi:10.2196/jmir.3439)
Supplement: Supplementary file 1 [file jmir_v16i8e191_app1.pdf]

Multimedia Appendix 1: MOOC Platforms

	Platforms
1	ALISON
2	Canvas.net
3	Coursera
4	CourseSites
5	Coursolve
6	Crypt-4-you
7	edX
8	France Universite Numerique
9	FutureLearn
10	Galileo Education Systems
11	Rwaq
12	Iversity
13	Miriada X
14	NovoEd
15	Open Learning
16	Open2Study
17	OpenHPI
18	OpenupEd
19	P2PUniversity
20	Saylor
21	Skynet
22	Udacity
23	Udemy
24	uneopen.com
25	UKeU (not in operation)
26	University of Miami Global
27	Veduca
2[illegible]	[illegible]
