# Supplementary material for: Massive Open Online Courses on Health and Medicine: Review
Source: J Med Internet Res. 2014 Aug 14;16(8):e191. doi: 10.2196/jmir.3439 (PMC4155756; doi:10.2196/jmir.3439)
Supplement: Supplementary file 2 [file jmir_v16i8e191_app2.pdf]

Multimedia Appendix 2: List of MOOCs considered in review

	MOOC Title	Platform	Start Date
1	International Health Systems	Canvas	07/01/2013
2	Introduction to Elements of Pain	Canvas	19/08/2013
3	Enhancing Patient Safety with Interprofessional Collaborative Practice	Canvas	26/08/2013
4	International Health Systems: Incorporating Sustainability Strategies	Canvas	23/09/2013
5	One Health One Medicine	Canvas	04/11/2013
6	Fundamentals of Human Nutrition	Coursera	22/01/2013
7	Health for All through Primary Care	Coursera	23/01/2013
8	An Introduction to the US Food System: Perspectives from Public Health	Coursera	23/01/2013
9	Contraception: Choices, Culture and Consequences	Coursera	28/01/2013
10	Health Informatics in the Cloud	Coursera	28/01/2013
11	Principles of Public Health	Coursera	28/01/2013
12	Nutrition for Health Promotion and Disease Prevention	Coursera	28/01/2013
13	The Social Context of Mental Health and Illness	Coursera	28/01/2013
14	Clinical Problem Solving	Coursera	11/02/2013
15	AIDS	Coursera	25/02/2013
16	"Pay Attention!!" ADHD Through the Lifespan	Coursera	18/03/2013
17	Health Policy and the Affordable Care Act	Coursera	25/03/2013
18	Medical Neuroscience	Coursera	08/04/2013
19	Healthcare Innovation and Entrepreneurship	Coursera	15/04/2013
20	Drug Discovery, Development & Commercialization	Coursera	19/04/2013
21	Community Change in Public Health	Coursera	22/04/2013
22	Genes and the Human Condition (From Behavior to Biotechnology)	Coursera	22/04/2013
23	Nutrition, Health and LifeStyle: issues and Insights	Coursera	06/05/2013
24	Child Nutrition and Cooking	Coursera	06/05/2013
25	Interprofessional Healthcare Informatics	Coursera	20/05/2013

26	Cardiac Arrest, Hypothermia, and Resuscitation Science	Coursera	20/05/2013
27	Rationing and Allocating Scarce Medical Resources	Coursera	20/05/2013
28	Health for All through Primary Care	Coursera	29/05/2013
29	Social Epidemiology	Coursera	31/05/2013
30	The Science of Safety in Healthcare	Coursera	03/06/2013
31	Nutrition for Health Promotion and Disease Prevention	Coursera	24/06/2013
32	Vaccine Trials: Methods and Best Practices	Coursera	24/06/2013
33	The Social Context of Mental Health and Illness	Coursera	24/06/2013
34	Nutrition and Physical Activity for Health	Coursera	15/07/2013
35	Case-Based Introduction to Biostatistics	Coursera	22/07/2013
36	Exercise Physiology: Understanding the Athlete Within	Coursera	22/07/2013
37	Virology I: How Viruses Work	Coursera	01/08/2013
38	Caries Management by Risk Assessment (CAMBRA)	Coursera	26/08/2013
39	An Introduction to Global Health	Coursera	02/09/2013
40	Vaccines	Coursera	03/09/2013
41	Contraception: Choices, Culture and Consequences	Coursera	09/09/2013
42	Generation Rx: The Science Behind Prescription Drug Abuse	Coursera	09/09/2013
43	Genetics and Society: A Course for Educators	Coursera	09/09/2013
44	Health Informatics in the Cloud	Coursera	16/09/2013
45	Data Management for Clinical Research	Coursera	16/09/2013
46	Health for All through Primary Care	Coursera	18/09/2013
47	Experimental Genome Science	Coursera	30/09/2013
48	Global Tuberculosis (TB) Clinical Management and Research	Coursera	30/09/2013
49	Global Health: Interdisciplinary Overview	Coursera	07/10/2013
50	Understanding and Improving the US Healthcare System	Coursera	07/10/2013
51	Introduction to Pharmacy	Coursera	11/10/2013
52	Care of Elders with Alzheimer's Disease and other Major Neurocognitive Disorders	Coursera	14/10/2013
53	Epidemics - the Dynamics of Infectious Diseases	Coursera	15/10/2013

54	Training and Learning Programs for Volunteer Community Health Workers	Coursera	21/10/2013
55	The New Nordic Diet - from Gastronomy to Health	Coursera	21/10/2013
56	Going out on a limb: Anatomy of the upper limb	Coursera	21/10/2013
57	Diabetese: Diagnosis, Treatment, and Opportunities	Coursera	28/10/2013
58	Genes and the Human Condition (From Behavior to Biotechnology)	Coursera	04/11/2013
59	Clinical Terminology for International and U.S. Students	Coursera	04/11/2013
60	Health Data Analytics MOOC	Coursera	05/11/2013
61	Major Depression in the Population: A Public Health Approach	Coursera	11/11/2013
62	Traditional Chineese Medinine and Chineese Culture	Coursera	01/12/2013
63	Introduction to Pharmaceutical Manufacturing Technologies	CourseSites	13/05/2013
64	So you want to work in the Pharmaceutical Industry	CourseSites	13/05/2013
65	Sustainable Healthy Diets	CourseSites	17/06/2013
66	Health Inequalities	CourseSites	15/07/2013
67	Perspectives on Disability	CourseSites	09/09/2013
68	Health Technology Assessment	CourseSites	28/10/2013
69	So you want to work in the Pharmaceutical Industry	CourseSites	25/11/2013
70	PH278x: Human Health and Global Enviornmental Change	EdX	15/05/2013
71	UT.4.01.x: Take Your Medicine - The Impact of Drug Development	EdX	16/09/2013
72	HSPH-HMS214x: Fundamentals of Clinical Trials	EdX	14/10/2013
73	PH201x: Health and Society	EdX	15/11/2013
74	Improving your image: Dental Photography in Practice	FutureLearn	02/12/2013
75	Enfermedades transfronterizas de los animales (Animal transboundary diseases)	Miriada X	25/02/2013
76	Retos de la agricultura y la alimentación en el siglo XXI (Challenges of Food and Agriculture in the XXI Century)	Miriada X	04/03/2013
77	La seguridad del paciente en el Domicilio	Miriada X	29/10/2013

	(Patient Safety in Home)		
78	Curso Práctico de Bioestadística con R (Primera parte) - biostatistics workshop with r (first part)	Miriada X	18/11/2013
79	Mobile Health Without Borders	NovoED	29/04/2013
80	Introduction to Nursing in Healthcare	Open2Study	22/04/2013
81	Food, Nutrition and Your Health	Open2Study	22/04/2013
82	Introduction to Nursing in Healthcare	Open2Study	27/05/2013
83	Food, Nutrition and Your Health	Open2Study	27/05/2013
84	Introduction to Nursing in Healthcare	Open2Study	01/07/2013
85	Food, Nutrition and Your Health	Open2Study	01/07/2013
86	Introduction to Nursing in Healthcare	Open2Study	09/09/2013
87	Food, Nutrition and Your Health	Open2Study	09/09/2013
88	Introduction to Nursing in Healthcare	Open2Study	14/10/2013
89	Food, Nutrition and Your Health	Open2Study	14/10/2013
90	Introduction to Nursing in Healthcare	Open2Study	18/11/2013
91	Food, Nutrition and Your Health	Open2Study	18/11/2013
92	The Human Body as a Machine	Open2Study	18/11/2013
93	Understanding Common Diseases	Open2Study	18/11/2013
94	HRP258: Statistics in Medicine	OpenEdX	11/06/2013
95	Mental wealth: know it and grow it	P2PUniversit y	28/10/2013
96	(Introduction to Psychiatry)	Rwaq	26/11/2013
97	Hippocrates Challenge	Stanford University VentureLab	15/05/2013
98	Understanding Dementia	University of Tasmania	29/07/2013
